# Meta-analysis reveals between-population differences affect the link between glucocorticoids and population health

**DOI:** 10.1093/conphys/coad005

**Published:** 2023-02-22

**Authors:** Levi Newediuk, Devon R Bath

**Affiliations:** Department of Biology, Memorial University, 45 Arctic Avenue, St. John's, Newfoundland A1B 3X9, Canada; Department of Ocean Sciences, Memorial University, 0 Marine Lab Road, St. John's, Newfoundland A1C 5S7, Canada

**Keywords:** Hormone, stressor, variation

## Abstract

Glucocorticoids are a popular tool for monitoring health of animal populations because they can increase with environmental stressors and can indicate chronic stress. However, individual responses to stressors create variation in the glucocorticoid–fitness relationship within populations. The inconsistency in this relationship calls into question the widespread use of glucocorticoids in conservation. We investigated the sources of variation in the glucocorticoid–fitness relationship by conducting a meta-analysis across a diverse set of species exposed to conservation-relevant stressors. We first quantified the extent to which studies inferred population health from glucocorticoids without first validating the glucocorticoid–fitness relationship in their own populations. We also tested whether population-level information like life history stage, sex and species longevity influenced the relationship between glucocorticoids and fitness. Finally, we tested for a universally consistent relationship between glucocorticoids and fitness across studies. We found more than half of peer-reviewed studies published between 2008 and 2022 inferred population health solely based on glucocorticoid levels. While life history stage explained some variation in the relationship between glucocorticoids and fitness, we found no consistent relationship between them. Much of the variation in the relationship could be the result of idiosyncratic characteristics of declining populations, such as unstable demographic structure, that coincided with large amounts of variation in glucocorticoid production. We suggest that conservation biologists capitalize on this variation in glucocorticoid production by declining populations by using the variance in glucocorticoid production as an early warning for declines in population health.

## Teaser

Glucocorticoid hormones are a popular health indicator for animal populations. We find the relationship between health and glucocorticoids is inconsistent. However, variation in glucocorticoids is prevalent and may characterize populations in decline. We suggest monitoring the variation in glucocorticoids within populations might be more useful than mean glucocorticoid levels.

## Introduction

Glucocorticoids are routinely used to make conservation decisions because of their ease of measurement and assumed relationship with physical condition and fitness. For example, a decade ago, hair cortisol concentration was identified as a highly useful metric for polar bear (*Ursus maritimus*) population health (Vongraven *et al.,* 2012), and since then, glucocorticoids have been linked to health effects precipitated by sea ice change (Boonstra *et al.,* 2020) and associated nutritional stress (Mislan *et al.,* 2016). In other species of conservation concern, including wood frogs (*Lithobates sylvatica,*Tennessen *et al.,* 2018) and Montagu's harrier (*Circus pygarus,*Rabdeau *et al.,* 2022), similar associations have been made between glucocorticoids and environmental and suspected threats to population health. Given their link to fitness, glucocorticoids have become a staple indicator for populations and species most vulnerable to environmental change. For these populations and species, consensus on the role of glucocorticoids in fitness outcomes is essential to their utility as a monitoring indicator for population health.

In a population health context, the prevailing perception is that glucocorticoids and fitness share a negative, linear relationship (Breuner *et al.,* 2008; Bonier *et al.,* 2009a). This relationship is supported by three potential mechanisms. First, environmental stressors, which manifest in eventual decline of populations, are followed by proximate stress responses by individuals (Bonier *et al.,* 2009a). In vertebrates, these responses are associated with glucocorticoids, which temporarily reallocate energy to surviving the stressor at the expense of reproductive effort (i.e. *cort trade-off hypothesis*, Patterson *et al.,* 2014). While reproductive success is compromised during stress responses, baseline glucocorticoids are often seasonally depressed, which lowers investment in survival to improve reproductive success (i.e. *cort-adaptation hypothesis*, Bonier *et al.,* 2009a). However, whether energy is allocated to survival or reproduction, population health is expected to decline with glucocorticoid production because relative exposure to more severe or frequent stressors reduces overall fitness (Breuner and Berk, 2019). Fitness is also thought to decline directly when physiological symptoms of chronic stress manifest from glucocorticoid overproduction (Kitaysky *et al.,* 2001). Thus, while glucocorticoid production is a beneficial response to stressors (e.g. Cote *et al.,* 2006), in a comparative context populations or species with higher glucocorticoid levels are usually considered less healthy.

Despite strong theoretical support for a negative glucocorticoid–fitness relationship, the direction and magnitude of the relationship among free-living animals is context dependent. Negative relationships between glucocorticoids and survival, for instance, are stronger in longer-lived species that accrue more negative effects from chronic stress (Schoenle *et al.,* 2021). Within species and populations, individual sex, age and reproductive maturity can influence the strength and direction of responses to the same stressors (Dantzer *et al.,* 2014). Individuals from the same population and cohort may exhibit different stress-coping phenotypes depending on early life experience and maternal exposure to stressors (Mommer and Bell, 2013). Stressors also elicit different responses over time, so that depending on when it is sampled, a single individual might produce widely different glucocorticoid levels (Taff *et al.,* 2018). For example, glucocorticoid production changed as individual house sparrow (*Passer domesticus*) body mass fluctuated over sampling periods (Baldan *et al.,* 2021) and as female albatross (*Diomedea exulans*) senesced (Angelier *et al.,* 2006). Indeed, reviews from the past decade failed to find a consistent glucocorticoid–fitness relationship across studies (Breuner *et al.,* 2008; Bonier *et al.,* 2009a; Dantzer *et al.,* 2014). The relationship is likely least consistent in free-living animals where glucocorticoids are measured using non-invasive samples, like feathers, hair and feces. Depending on which individuals are sampled and when, the glucocorticoid–fitness relationship might change. This inconsistency calls into question the use of glucocorticoids as a monitoring indicator for population health.

A focus on the direction of the glucocorticoid–fitness relationship among conservation biologists has directed attention away from inconsistency in the relationship. We contend that for glucocorticoids to be useful as an indicator of population health, focus should be redirected to the variability that causes of different glucocorticoid–fitness relationships. The primary reason for understanding variability is that mean levels of glucocorticoids and fitness do not reflect the breadth of variation in glucocorticoid production and fitness within populations. For example, a stressor might lower mean fitness within a population. However, an expected negative relationship between glucocorticoids and fitness might be absent if among-individual variation in glucocorticoid production within the population overlaps with mean glucocorticoid production in another population. Alternatively, if two populations are exposed to a detrimental stressor, both may manifest negative fitness consequences. These negative consequences may persist even if one population has prior experience with the stressor and produces fewer glucocorticoids because it is habituated. If glucocorticoids reallocate energy to survival and away from reproduction, conservation biologists might detect either a positive or negative relationship between glucocorticoids and reproduction, depending on which aspect of fitness is measured. In these contexts, the amount of variation in glucocorticoid production within a population might be a more useful measure because it reflects the diversity of individuals and their history of exposure to stressors.

We conducted a systematic review and meta-analysis of the relationship between glucocorticoids and fitness measures in animals exposed to conservation-relevant stressors. We first highlight the relevance of this question to conservation by quantifying what proportion of recent studies make conservation recommendations after relying on glucocorticoids as a proxy for population health. We then separately quantify the effect of stressors on glucocorticoid production and fitness measures, and the relationship between the two, to test the assumption that the relationship between glucocorticoid production and fitness is universally negative. We also test the contribution of life-history stage, sex and species longevity to variability in the glucocorticoid–fitness relationship. We conclude by discussing how the assumption of this negative relationship, given the many sources of variability, may mislead conservation efforts. Finally, we recommend a revised approach to monitoring variation in glucocorticoid production to improve the utility of glucocorticoids as a conservation tool.

## Methods

### Systematic review

We first conducted a systematic review to assess the degree to which studies conducted in conservation physiology relied on glucocorticoids as a proxy for population health. Three major reviews were published in 2008 and 2009, all of which challenged the idea of a universally negative relationship between glucocorticoids and fitness (Breuner *et al.,* 2008; Busch and Hayward, 2009; Bonier *et al.,* 2009a). Another conservation-focussed perspective was published several years later (Dantzer *et al.,* 2014). We expected conservation physiology studies with access to these four influential reviews—i.e. those published post-2008—would be less likely to make inferences about glucocorticoid levels in their study populations without first testing the relationship between glucocorticoids and a measure of fitness. Thus, we searched for studies published between 2008 and 2022. See Supplementary File 1 S2 for a full description of the search query. When a study measured glucocorticoids, we recorded whether they also measured fitness, and whether they implied glucocorticoid levels were related to population health. We categorized papers into four subgroups: those which did not measure any metric associated with fitness but made inferences about population health; those which did measure some aspect associated with fitness and made inferences about population health; those that measured some aspect associated with fitness but did not make any inferences about population health; and those that neither measured any aspect associated with fitness nor made inferences about population health.

### Search strategy and screening

**Figure 1:**
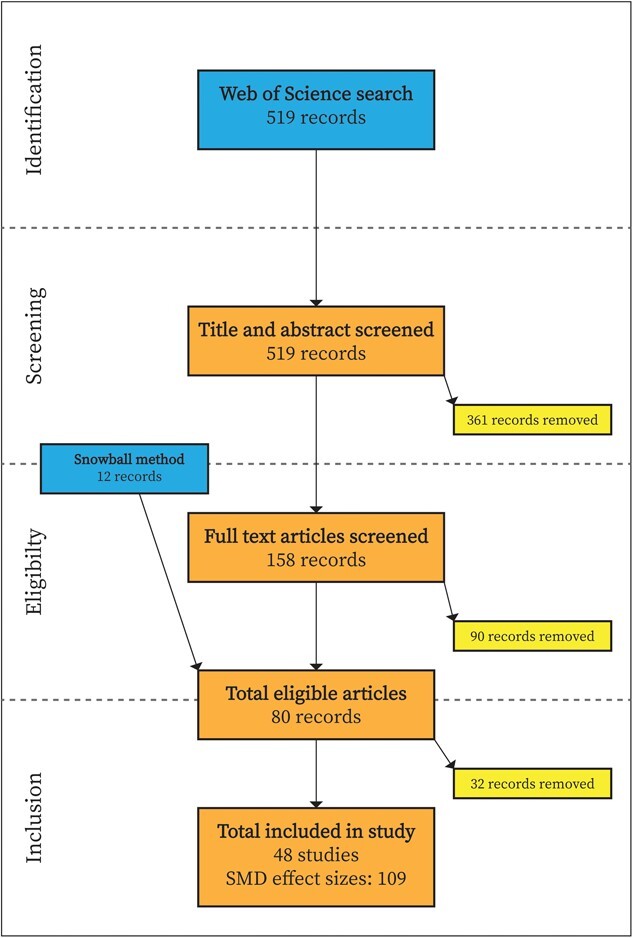
PRISMA diagram for study eligibility. Snowball method involved searching for relevant studies missed in the initial search from the reference sections of 68 eligible studies. SMD, standardized mean difference.

We conducted our meta-analysis following PRISMA reporting guidelines (Liberati *et al.,* 2009; O'Dea *et al.,* 2021; Fig. 1). We systematically searched the *web of science core collection* (WOS) database with a keyword set to target studies that measured glucocorticoid production (e.g. ‘cortisol’, ‘glucocorticoids’, ‘corticosterone’, ‘FGM’) and fitness metrics (e.g. ‘reproductive success’, ‘survival’, ‘body condition’) in response to stressors related to either food limitation or predation risk (e.g. ‘predation risk’, ‘food limitation’, ‘density’). Fitness, i.e. the average genetic contribution of an individual to future generations, is best measured using direct measures of either short-term survival or reproductive success. However, both survival and reproductive success are difficult to quantify in natural contexts. Thus, we also considered metrics like body condition, on which many studies in free-living animals rely as a proxy for fitness. We limited our search to stressors related to food limitation and predation risk with the assumption that from the perspective of an individual animal, all stressors are ultimately either directly related to food limitation or predation risk, or to competition for food resources and safety from predation risk. For example, glucocorticoid production increases when selective timber harvest produces forest gaps that elevate perceived predation risk (Leshyk *et al.,* 2013). Similarly, resource availability differences between forest fragments change conspecific density and encourage competition for food (Gabriel *et al.,* 2018). See Supplementary File 1 S1 & S2 for our screening procedures and full search query strategy.

We found a total of 519 studies using our search query. Since we were interested in the effects of exogenous stressors on fitness, we excluded any studies that manipulated glucocorticoid levels by dexamethasone challenge. We only included studies if they either measured or manipulated an environmental stressor or subjected animals to an experimental stressor in a laboratory situation. Finally, we considered only studies published after the Breuner *et al.* (2008) review since we assumed those authors, and the authors of other reviews published shortly thereafter (Busch and Hayward, 2009; Bonier *et al.,* 2009a; Dantzer *et al.,* 2014), thoroughly reviewed literature published prior to 2008. We used our criteria to exclude 385 studies based on title and abstract, finally retrieving 158 full-text studies published between January 2008 and September 2022.

### Study selection

We attempted to extract data from 80 studies after excluding studies that did not meet our criteria upon full-text review. Our criteria included both experimental and observational studies that measured glucocorticoid (cortisol, corticosterone, fecal metabolites) production in response to at least two different levels of a stressor, or one group exposed to a stressor paired with a control group. We included studies that measured either baseline glucocorticoids (i.e. circulating glucocorticoids measured within 3 minutes of capture, Romero and Reed, 2005), acute glucocorticoid production (i.e. circulating glucocorticoids following the 3 minutes after exposure to a stressor, Romero and Reed, 2005) or integrated glucocorticoids (e.g. fecal glucocorticoid metabolites, yolk corticosterone, whole body cortisol). We also required a measure of fitness for each level of stressor representing either survival, immune function, body condition or reproductive success. We found 68 of the 158 full-text studies we considered met our criteria for data extraction. We also found an additional 12 suitable studies we missed in our initial search using a ‘snowballing’ method, i.e. we scanned the reference sections of the 68 eligible full-text studies for relevant references.

To compare glucocorticoid production and fitness measures between our two stressor levels, we calculated standardized mean differences using the package ‘metafor’ (Viechtbauer, 2010) in R (R Core Team, 2022). We focussed on differences in glucocorticoid production and fitness measures between animals exposed to a stressor and those either unexposed or exposed to a lower level of the stressor. If animals were exposed to more than two levels of a stressor, we only considered the highest and lowest levels. In cases where a single group was exposed to a stressor that changed over time or effects of the stressor were measured more than once, we considered only the first and last times glucocorticoids and fitness were measured. When multiple measures of a single fitness type were reported, we used one from each category, prioritizing the measure most clearly related to fitness (e.g. fledging success instead of number of eggs; body mass instead of body size). We also reversed fitness measurements if a larger fitness value represented lower fitness, or vice versa (e.g. muscle enzyme activity). We collected mean glucocorticoid levels, mean fitness levels and a measure of variability and sample size at each stressor level directly from manuscript text, tables or supplementary data files where possible. When data were present only in figures, we extracted means and measures of variability using ‘ImageJ’ (National Institutes of Health, USA). We split effect size calculations by life history stage and sex (i.e. juveniles and adults, males and females) where both glucocorticoids and fitness measures were measured separately. If studies included only one measure of either glucocorticoids or fitness, we combined groups by calculating the grand mean and pooled variance across life history stages and sexes. When sample sizes were reported as a range, we used the lowest sample size.

### Meta-analytic models

We individually tested the mean effect of stressors on both glucocorticoids and fitness measures by fitting two intercept-only meta-analytic models using the function ‘rma.mv’ in ‘metafor’. We also fit six models with moderators to test the effects of life history stage, longevity and sex on both glucocorticoids and fitness measures. We obtained longevity data from the Animal Ageing and Longevity Database (AnAge). In some contexts, glucocorticoids are thought to direct energy towards survival (Wingfield and Sapolsky, 2003), while in other contexts, they support reproduction (Bonier *et al.,* 2009a). To control for different relationships between glucocorticoids and fitness measures, we included fitness type (body condition, immune function, survival or reproductive success) as a random effect in all meta-analytic models testing fitness effect sizes across stressor levels. Whether glucocorticoids support survival or reproductive success is also species-specific (Schoenle *et al.,* 2021), so we also included species as a random effect in all models. We included stressor type as a random effect to control for potentially different responses to predation risk and food limitation. Finally, we included study ID as a random effect. We fit all models using the ‘metafor’ package in R.

### Bivariate models

To test the overall relationship between fitness measures and glucocorticoids across studies, we fit a bivariate mixed model using Markov Chain Monte Carlo (MCMC) with the package ‘MCMCglmm’ (Hadfield, 2010) in R. We included fitness type, stressor type and life history stage as fixed effects. Finally, we included study ID as a random effect. We used weakly informative parameter-expanded priors and scaled both response variables prior to modelling (Houslay and Wilson, 2017). We ran our model using 420 000 iterations, including 20 000 burn-in period and 100 thinning intervals. Bivariate models use the covariance between response variables—in our case glucocorticoids and fitness measures—to account for sampling error. Since none of our studies reported covariance and we did not have access to raw glucocorticoid and fitness data with which to estimate covariance, we specified the effect size variance of glucocorticoids and fitness measures in the ‘mev’ argument in the MCMCglmm function.

### Publication bias

We tested for the existence of publication bias among our studies using funnel plots created using the ‘funnel’ function in ‘metafor’. We also evaluated the severity of any publication bias using the fail-safe number (FSN). Publication bias is thought to arise when studies lacking significant results remain unpublished (i.e. the file-drawer problem, Rosenberg, 2005). Asymmetry of funnel plots, which compare effect sizes across a measure of effect size precision, provides a visual cue for bias. Positively skewed effect sizes suggest the sample of studies included in the meta-analysis might overreport positive results, positively biasing mean effect sizes (Schielzeth and Nakagawa, 2022). To statistically test for bias related to small sample sizes, we also re-fit our meta-analytic models with an additional moderator for the inverse of the sample size (Moran *et al.,* 2021). Finally, we evaluated the robustness of our results by calculating the FSN, or the number of nonsignificant and unpublished studies that would need to be added to our meta-analysis to render our statistically significant results nonsignificant (Rosenberg, 2005). Complete data and R code to replicate analyses are available at https://doi.org/10.5281/zenodo.7586897.

## Results

### Systematic review

**Figure 2:**
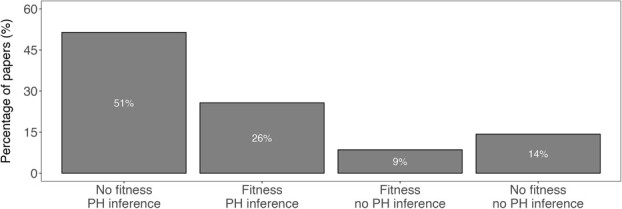
Results from systematic review on recent conservation physiology studies relying on glucocorticoids as a proxy for population health. We compared whether studies which both measured glucocorticoid production and made inferences about the implications of glucocorticoid production on population health (PH) also measured fitness.

In our systematic review, we found 35 studies published since 2008 that used glucocorticoids as proxy for population health. We found over half or 18 of 35 studies wrote about fitness implications of glucocorticoids without first validating the relationship between glucocorticoids and fitness in their populations (Fig. 2). Of the 12 studies that did validate the relationship between glucocorticoids and fitness, 9 also discussed the implications of glucocorticoids for population health. A final 5 of the 35 studies we found using our search criteria neither measured fitness nor implied any relationship between glucocorticoids and population health.

### Meta-analysis data characteristics

We included 48 studies in our meta-analysis after excluding 32 for which missing data (i.e. means, measures of variability or sample sizes) prevented us from calculating standardized mean differences. Effect size data are available in Data File 1. We calculated 109 effect sizes in total, 78 of which were from experimental studies and 31 from observational studies. Most studies used an invasive method to sample glucocorticoids (e.g. drawing blood), but 13 of 109 effect sizes were from studies that non-invasively sampled glucocorticoids from the field or environment (e.g. collected fecal samples). Field studies constituted 43 of the 109 effect sizes, and the remaining effect sizes were from studies conducted in the laboratory. We found neither sample type, invasiveness, nor study location accounted for a significant amount of heterogeneity in glucocorticoid effect sizes (Supplementary File 1 S5). Stressors related to food availability were usually directly measured or experimentally manipulated (e.g. food restriction, manipulation of population density), while most predation risk stressors were proxies or based on the environment (e.g. local predator abundance, traffic noise, etc.). Of the 109 effect sizes, 96 were comparisons between single measures of glucocorticoid and fitness from two different populations and 13 compared changes in glucocorticoids and fitness within the same populations over time. The studies we included were taxonomically diverse, representing 41 species. Of these species, 36 effect sizes were from birds, 35 were from fish, 28 from mammals, 6 from amphibians and 4 from reptiles. Only 7 effect sizes either did not report or combined life history stages, and otherwise were approximately equally represented by 50 adult groups and 52 juvenile groups. Males and females were combined in 81 effect sizes and there were 29 effect sizes specific to either females or males. Metadata from all studies are available in Data File 2.

**Figure 3:**
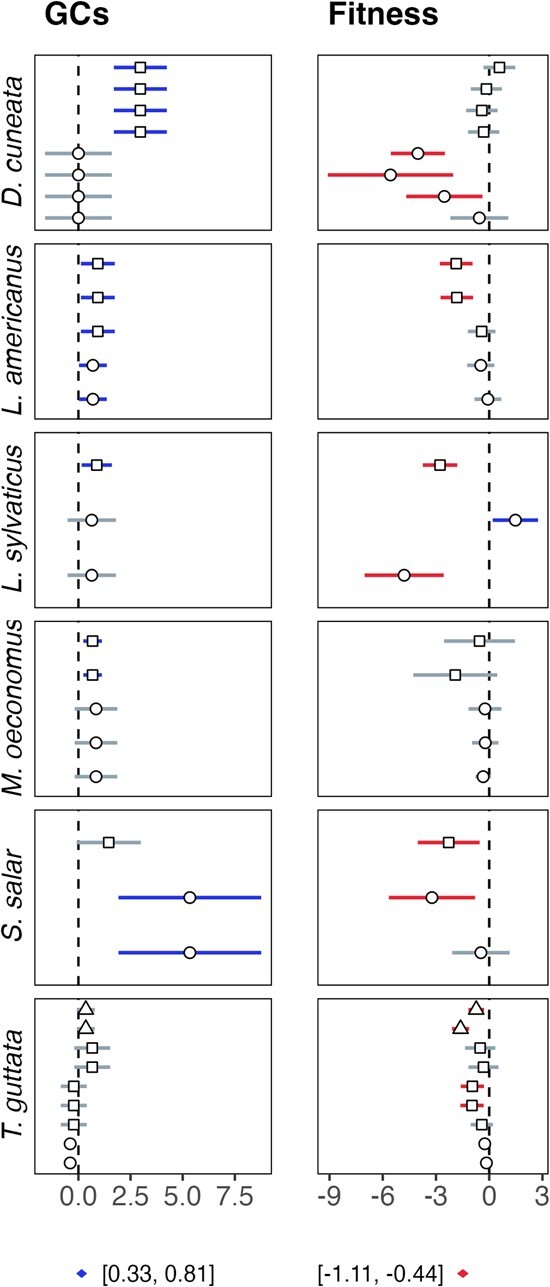
Mean effect sizes (± 95% confidence intervals) for glucocorticoid production and fitness in response to stressors for six example species (*Dicologlossa cuneata, Lepus americanus, Lithobates sylvaticus, Microtus oeconomus, Salmo salar* and *Taeniopygia guttata*) used in multiple studies. Blue indicates a positive effect size with confidence interval above zero, red indicates a negative effect size below zero, and grey indicates an effect size that crosses zero. Different symbols in each panel indicate different studies. Symbols below panels show the grand mean effect sizes [± 95% confidence intervals] for glucocorticoids and fitness across all 48 studies, indicating glucocorticoid production generally increases, and fitness decreases, in response to stressors. However, the direction of the glucocorticoid–fitness relationship is inconsistent even between populations belonging to the same species.

### Meta-analytic models

Glucocorticoid production and fitness measures differed in response to stressors and according to context. Exposure to stressors generally increased mean glucocorticoid levels and decreased mean measures of fitness (Fig. 3; Supplementary File 1 S3). Juvenile fitness measures decreased more than adults after exposure to stressors, and in response to stressors, juveniles also produced more glucocorticoids than adults (Table 1). Males and females differed neither in terms of glucocorticoid production nor fitness measures in response to stressors (Table 1), and sex did not account for a significant amount of moderator heterogeneity (glucocorticoids: Q_M_ = 0.83, *P* = 0.36; fitness measures: Q_M_ = 0.039, *P* = 0.84). Life history accounted for a significant amount of heterogeneity in both glucocorticoid production (Q_M_ = 5.61, *P* = 0.0179) and fitness measures (Q_M_ = 13.91, *P* = 0.0003). However, species longevity affected neither fitness measures nor glucocorticoid production (Table 1). There was significant residual heterogeneity in all models, suggesting a large amount of variation among studies even after accounting for moderator effects (Table 1). Between-study variation was large even among different studies on the same species (Fig. 3).

**Table 1:** Pooled mean effect sizes and moderator estimates from meta-regression mixed-effects models testing effects of stressor exposure on glucocorticoid (GC) production and fitness

Moderator	ß (95% CI)	Q-test for heterogeneity (df)	N studies/effect sizes
—			
GC	**0.58 (0.29–0.87)**	**435.86 (106)**	47/107
Fitness	**−0.78 (−1.11 to −0.44)**	**804.20 (106)**	
Sex: male			
GC	−0.18 (−0.56 to 0.21)	**123.39 (26)**	12/27
Fitness	0.04 (−0.36 to 0.44)	**178.19 (26)**	
Age: juvenile			
GC	**0.52 (0.09–0.94)**	**374.98 (98)**	43/99
Fitness	**−1.01 (−1.55 to −0.46)**	**627.84 (98)**	
Longevity			
GC	0.01 (−0.02 to 0.04)	**161.40 (46)**	27/47
Fitness	−0.04 (−0.09 to 0.01)	**408.38 (46)**	

All models include random effects for species and individual effect size nested in individual study. GC ~ ß models also include a random effect for stressor type (predation risk, food limitation, density) and fitness ~ ß models include a random effect for fitness type (reproductive success, survival, immune response, body condition). Reference categories for sex and age moderators are female and adult, respectively. Bolded entries indicate significant effects (*P* < 0.05)

### Bivariate models

**Figure 4:**
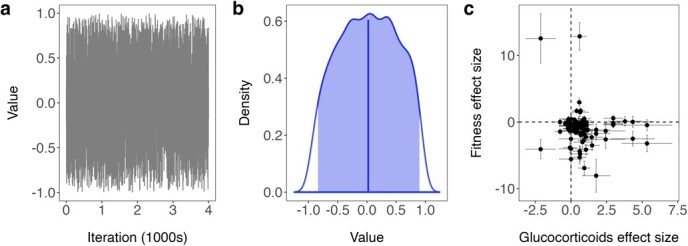
Correlation between glucocorticoid and fitness effect sizes. In (a), the trace plot of the bivariate model indicates adequate exploration of the posterior distribution. The highest posterior density interval (shaded region and posterior mean at vertical line) of the posterior distribution encompasses zero (b), indicating no significant correlation between glucocorticoid levels and fitness. Finally, a scatter plot of raw effect sizes (± SE) illustrates a weak negative relationship between glucocorticoid production and fitness in response to stressors with large within-study variability in both measures (c).

There was no overall correlation between glucocorticoids and fitness measures after controlling for within-study variation using a bivariate model. The model trace plots indicated adequate exploration of the posterior distribution, i.e. no obvious pattern in the Markov chain (Fig. 4a). The posterior distribution crossed zero, indicating no relationship between glucocorticoids and fitness measures (Fig. 4b). While many fitness effect sizes were negative and glucocorticoid effect sizes were positive, the standard errors of most effect sizes were large, suggesting variability might have contributed to the absence of a relationship between them (Fig. 4c).

### Publication bias

There was some evidence of publication bias among our studies. While funnel plots did not suggest publication bias in fitness effect sizes, glucocorticoid effect sizes skewed more positively than expected (Supplementary File 1 S4). Similarly, our statistical test, i.e. meta-analytic models with an additional moderator for the square root of the inverse sample size, found no publication bias in terms of fitness effect sizes (effect size estimate, −1.54; 95% confidence interval [CI], −3.78 to 0.71]), but effect sizes were biased towards positive effects of stressors on glucocorticoids (1.61; 95% CI, 0.08–3.14). The FSNs were 2855 for our fitness model and 5467 for our glucocorticoid model, suggesting a large number of unpublished non-significant results would be required to change our results.

## Discussion

We found exposure to conservation-relevant stressors both increased mean glucocorticoid production and decreased mean measures of fitness, consistent with the negative relationship between glucocorticoids and fitness espoused by conservation biologists (e.g. Fig. 2). However, we did not find a consistent relationship between glucocorticoids and fitness measures across studies. For some study populations, stressors that increased glucocorticoid production also increased fitness measures. Similarly, stressors that compromised measures of fitness sometimes coincided with decreases in glucocorticoid production. Both situations imply a positive relationship between glucocorticoids and fitness. Unexpected relationships between glucocorticoids and fitness are sometimes attributed to seasonal or life-history stage transitions, which are often marked by changes in glucocorticoid production (Romero, 2002; Crespi *et al.,* 2013). Consistent with this explanation, we found some of our substantial between-study variation in glucocorticoid production was explained by life-history stage. However, life-history stage did not account for all variation in glucocorticoid production. Moreover, neither glucocorticoid production nor fitness measures were explained by sex of individuals measured, taxonomic group or species longevity (Table 1). In fact, even study populations belonging to the same species exhibited widely different glucocorticoid–fitness relationships (Fig. 4). Furthermore, our publication bias tests suggested a slight bias towards publishing studies with higher glucocorticoid production in response to stressors, i.e. those studies supporting the expected negative relationship between glucocorticoids and fitness. Despite the emphasis on this negative relationship in the literature, the widespread use of mean glucocorticoid levels as an indicator of population health is tenuous given the amount of variation in the relationship.

Much of the variation we found in both glucocorticoid production and fitness measures manifested from differences between studies. While high between-study heterogeneity is more acceptable in ecology and evolution meta-analyses compared to other fields (O’Dea *et al.,* 2021), we still found significant heterogeneity in all meta-analytic models (Table 1). Even life history stage, which accounted for a significant portion of effect size variation among the moderators we tested, still resulted in a model with high residual heterogeneity (Table 1). While in some cases heterogeneity can result from methodological differences like field versus laboratory settings or sample type (Sheriff *et al.,* 2010; Rowland and Toth, 2019), we suggest most of the heterogeneity between studies in our meta-analysis likely arose from population-level differences. For example, in one tree swallow (*Tachycineta bicolor*) population, late-season glucocorticoid production improved offspring fitness during provisioning (Bonier *et al.,* 2009b). As of our last WOS search in late 2022, this study had been cited over 200 times. Some cited the importance of elevated glucocorticoids for fitness in their own tree swallow populations (e.g. Injaian *et al.,* 2019). However, another study using a different tree swallow population several years later found no relationship between glucocorticoids and fitness (Madliger and Love, 2016). The glucocorticoid–fitness relationship from a single population often serves as a benchmark for future studies on the species. Indeed, we found more than 50% of studies that used glucocorticoid levels to make conservation recommendations relied on the glucocorticoid–fitness relationship reported by a previous study (Fig. 2). However, the tree swallow example suggests generalizing the glucocorticoid–fitness relationship across species may be inappropriate, particularly when data come from a single population.

Before drawing inferences about population health from glucocorticoid levels in their own populations, studies should consider the factors that shape glucocorticoid production in other populations. We tested whether either glucocorticoid levels or fitness measures varied with life history stage, sex or species longevity. While we did not find that sex contributed to variation in either glucocorticoids or fitness measures among the studies included in our meta-analysis, previous studies found males produce more glucocorticoids than females (e.g. Lafferty *et al.,* 2015; Mugabo *et al.,* 2017). We also found no relationship between glucocorticoid production and species longevity in our meta-analysis. However, glucocorticoid production tends to decline with age (e.g. Heidinger *et al.,* 2006; Kelm *et al.,* 2016), which aligns with our finding that juveniles produced more glucocorticoids than adults. Theoretically, glucocorticoid levels might vary between two populations because of age structure or sex ratio even if both populations experience the same stressors. Thus, glucocorticoid levels might simply reflect demographic differences between populations, or among the subset of individuals sampled, rather than differences in population health.

Past exposure of populations to stressors can also complicate the relationship between population health and glucocorticoid production. For example, downregulating glucocorticoid production is sometimes a strategy used by animals facing chronic stressors to avoid pathological levels of glucocorticoids (Rich and Romero, 2005). Indeed, stressors caused glucocorticoid production to decrease in eight studies or approximately 20% of all studies in our meta-analysis (Fig. 4). In two of these studies fitness measures also decreased in response to the stressor, suggesting lower glucocorticoid levels are sometimes linked to chronic stress. The link between chronic stress and downregulation of glucocorticoid production means that lower glucocorticoid levels might also be characteristic of populations in poorer health. For example, zebra finches exposed to traffic noise had lower glucocorticoids and slower-growing offspring (Zollinger *et al.,* 2019). However, glucocorticoid downregulation can also indicate acclimation to a chronic stressor that no longer affects fitness. For example, traffic noise produced a stress response and compromised immune responses in a wood frog (*Rana sylvatica*) population with no previous exposure to the noise, but a different population with a history of traffic noise exposure both produced fewer glucocorticoids and avoided disruptions to immune function (Tennessen *et al.,* 2018). The complexity of glucocorticoid production, therefore, complicates between-population comparisons without information about past exposure to stressors.

Given population-specific relationships between glucocorticoids, stressors and fitness, how useful are glucocorticoids as a generalized indicator for population health? Our results suggest information about individuals might provide the context needed to link glucocorticoids to population health because demographic information, for example, contextualizes glucocorticoid production. This context dependence has prompted others to urge understanding of the factors responsible for unexpected glucocorticoid–fitness relationships (Madliger and Love, 2016). However, the demographic data required to contextualize glucocorticoid–fitness relationships are lacking for many vertebrate populations (Conde *et al.,* 2019). Crucially, populations with the least available data are often those of greatest conservation concern (Good *et al.,* 2006). The ethics of capturing species of conservation concern, in addition to the rarity of populations, often limits glucocorticoid measures to non-invasive samples. However, non-invasive samples are characterized by differences in production and metabolism of glucocorticoids both among individuals and over time (Palme, 2019). Thus, the species for which non-invasive samples might be the only data available are also the species for which glucocorticoids are likely the most useful monitors of health. Paradoxically, these species might also exhibit the most variation in glucocorticoid production, and consequently, the most unexplained variation in their glucocorticoid–fitness relationships.

We suggest variation in glucocorticoid production might provide an alternative way to monitor population health. Rather than assessing population health based on the mean glucocorticoid levels of populations, we suggest using the variation in glucocorticoid production among individuals and over time to predict when populations are at risk of decline. We found between-population variability in glucocorticoid–fitness relationships stemmed in part from differences in glucocorticoid production between adults and juveniles. Consequently, populations whose demographic structures vary over time may also be most variable in terms of glucocorticoid levels. Unstable demographic structures also coincide with size fluctuations in declining populations (Jackson *et al.,* 2020), suggesting glucocorticoid production might be most variable in the least healthy populations. Population variation in glucocorticoid production also arises from individual differences in stress coping styles (Koolhaas *et al.,* 2010), which might characterize populations dealing with long-term stressors. Thus, populations facing the most severe stressors, and associated negative effects on health, may also exhibit the most variability in glucocorticoid production. We suggest comparing the variation in glucocorticoid production between populations, rather than average levels of glucocorticoid production, might be the most robust monitor for population health.

## Conclusion

Glucocorticoids are considered a convenient tool for measuring population health. However, the context dependence of glucocorticoid production introduces variation into the relationship between glucocorticoids and fitness. We showed that much of the variation in the relationship between fitness measures and glucocorticoids stems from variation in glucocorticoid production between populations. This between-population variation is rooted in population-specific characteristics like demographic structure and history of exposure to stressors. While variation poses a problem for generalizing the glucocorticoid–fitness relationship across species, it might parallel the variation in other population characteristics like age structure that are directly related to population health. We contend that conservation can still benefit from measuring glucocorticoids. Instead of searching for a consistent direction in the glucocorticoid–fitness relationship, we suggest the variation in glucocorticoid levels might provide more clues about population health.

## Funding

No support was provided.

## Supplementary data

Supplementary material is available at *Conservation Physiology* online.

## Data availability

Data are available as Data Files 1 & 2. Complete data and R code to replicate analyses are available at https://doi.org/10.5281/zenodo.7586897.

## Supplementary Material

Web_Material_coad005
