# Supplementary material for: Meta-analysis reveals between-population differences affect the link between glucocorticoids and population health
Source: Conserv Physiol. 2023 Feb 22;11(1):coad005. doi: 10.1093/conphys/coad005 (PMC9945071; doi:10.1093/conphys/coad005)
Supplement: Web_Material_coad005 [file web_material_coad005.zip › Supplementary1-2.pdf]

Supplementary material for: Meta-analysis reveals between-population differences affect the link between glucocorticoids and population health

Levi Newediuk, Devon R. Bath

Supplementary S1. Decision tree for eligibility at the extraction stage (Fig. S1)

Supplementary S2. Web of Science query to identify studies for screening

Supplementary S3. Forest plots for all fitness effect sizes (Fig. S2) and glucocorticoid effect sizes (Fig. S4) included in meta-analysis

Supplementary S4. Funnel plots for publication bias (Figs. S4–S11)

Supplementary S5. Effects of sample type and sampling invasiveness on heterogeneity in glucocorticoid effect sizes (Table S1).

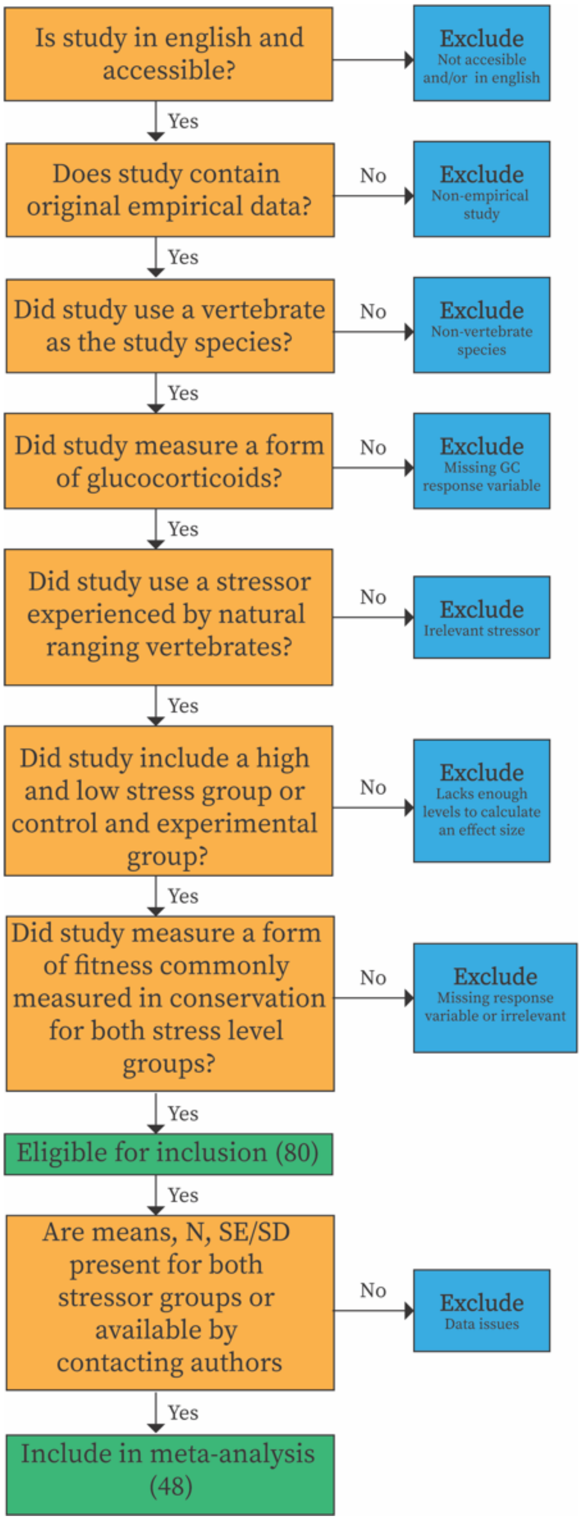

Fig. S1. Decision tree for study inclusion at the data extraction stage. Parentheses represent the number of studies included at the extraction stage and those from which we extracted data.

Supplementary S2. Web of Science search query to identify studies for systematic review and screening in meta-analysis

To identify studies for our systematic review, we searched Web of Science for peer-reviewed English-language studies using the following query:

Web of Science core collection: (((ALL=(“glucocorticoid”)) AND ALL=("fitness”)) AND ALL=(“conservation”) Date range(yyyy-mm-dd): 2008-01-01 to 2022-09-01

Our search yielded 62 records. After excluding studies that did not include original data (i.e., review articles) and those that did not measure some form of glucocorticoid levels, we considered 35 studies.

To identify studies for our meta-analysis, we searched Web of Science for peer-reviewed English-language studies using the following query:

Web of Science core collection: (((ALL=(“glucocorticoid*" OR “FGM" OR “corticosterone" OR "cortisol")) AND ALL=("reproductive success” OR “survival" OR "fitness”)) AND ALL=(“vigilance" OR "predation risk" OR “density" OR "food limitation" OR "nutritional stress" OR "territoriality" OR "aggressive interactions”)Date range(yyyy-mm-dd): 2008-01-01 to 2022-09-01

We included the first set of terms to identify studies that included some measure of glucocorticoid levels. The second set of search terms identified only the studies that also measured some form of fitness, which we could relate to population health. We used the third set of search terms to identify conservation-relevant stressors.

Supplementary S3. Forest plots for all fitness effect sizes (Fig. S2) and glucocorticoid effect sizes (Fig. S3) included in meta-analysis

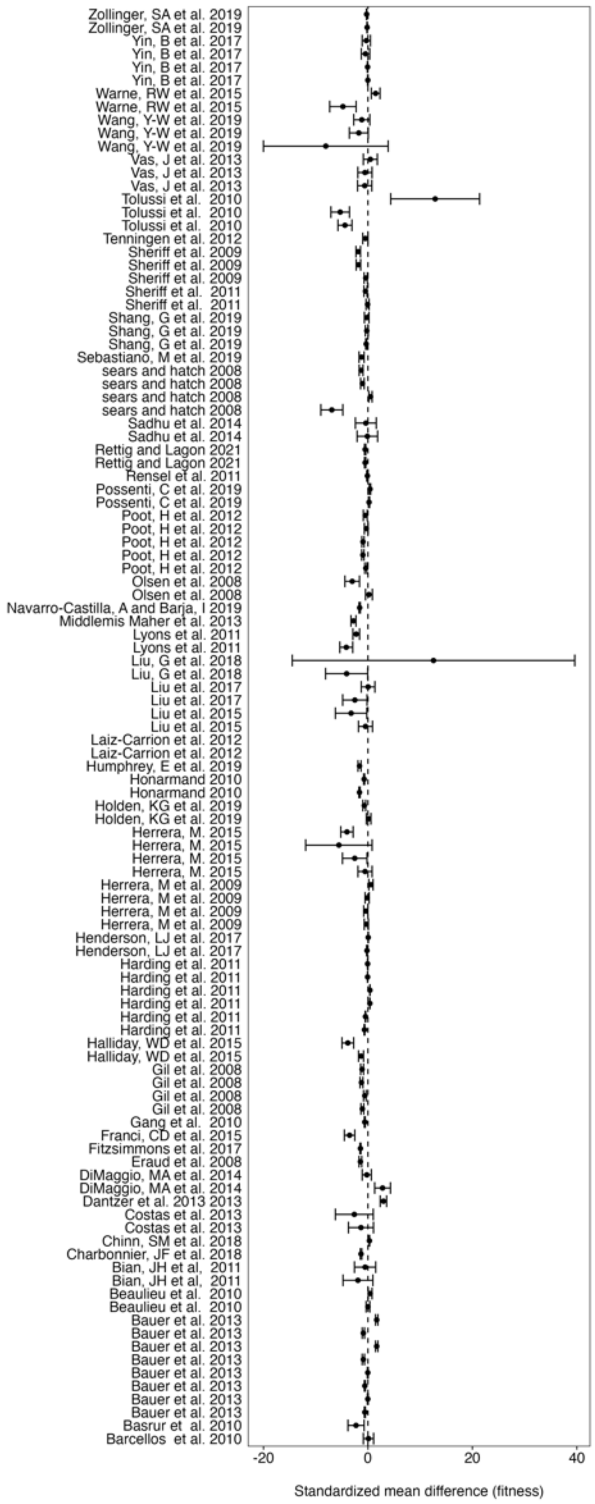

Fig. S2. Forest plot showing fitness effect sizes for all studies included in meta-analysis. Black points are mean effect sizes and bars are 95% confidence intervals of the effect size.

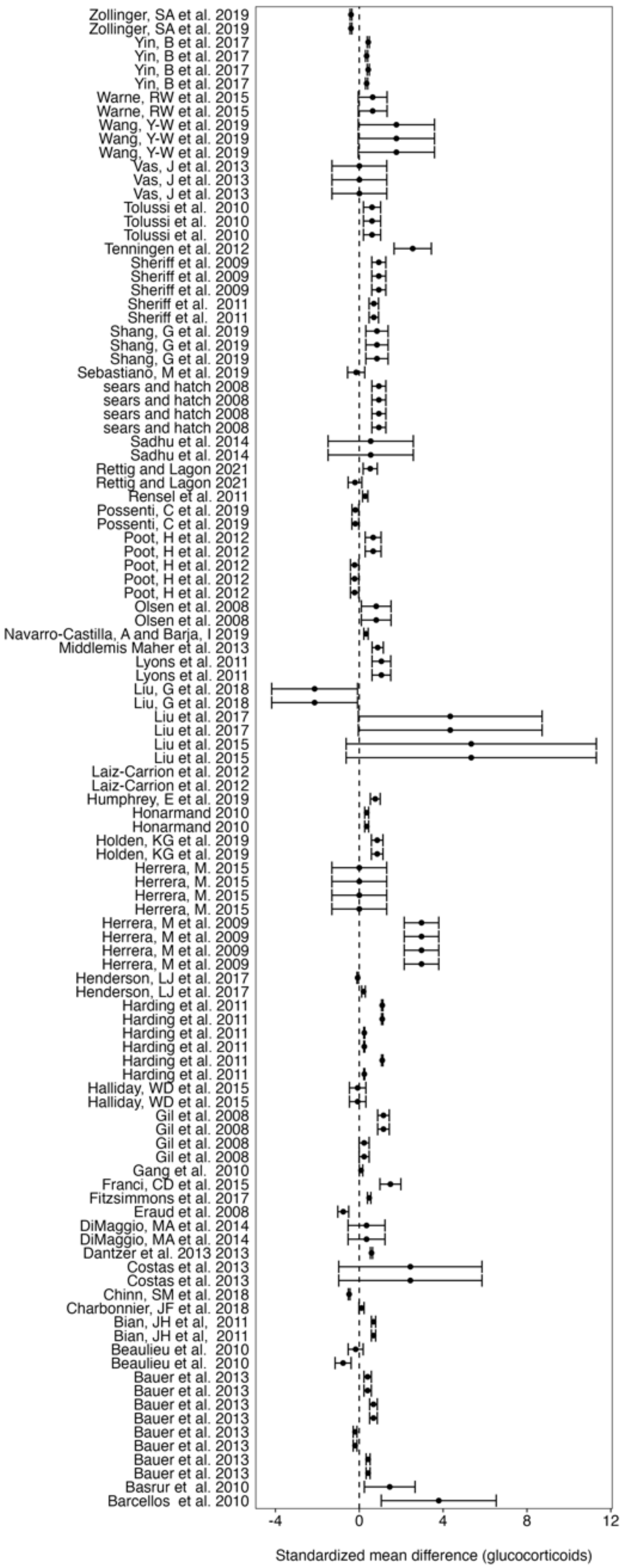

Fig. S3. Forest plot showing glucocorticoid effect sizes for all studies included in meta-analysis. Black points are mean effect sizes and bars are 95% confidence intervals of the effect size.

Supplementary S4. Funnel plots for publication bias (Figs. S4–S11)

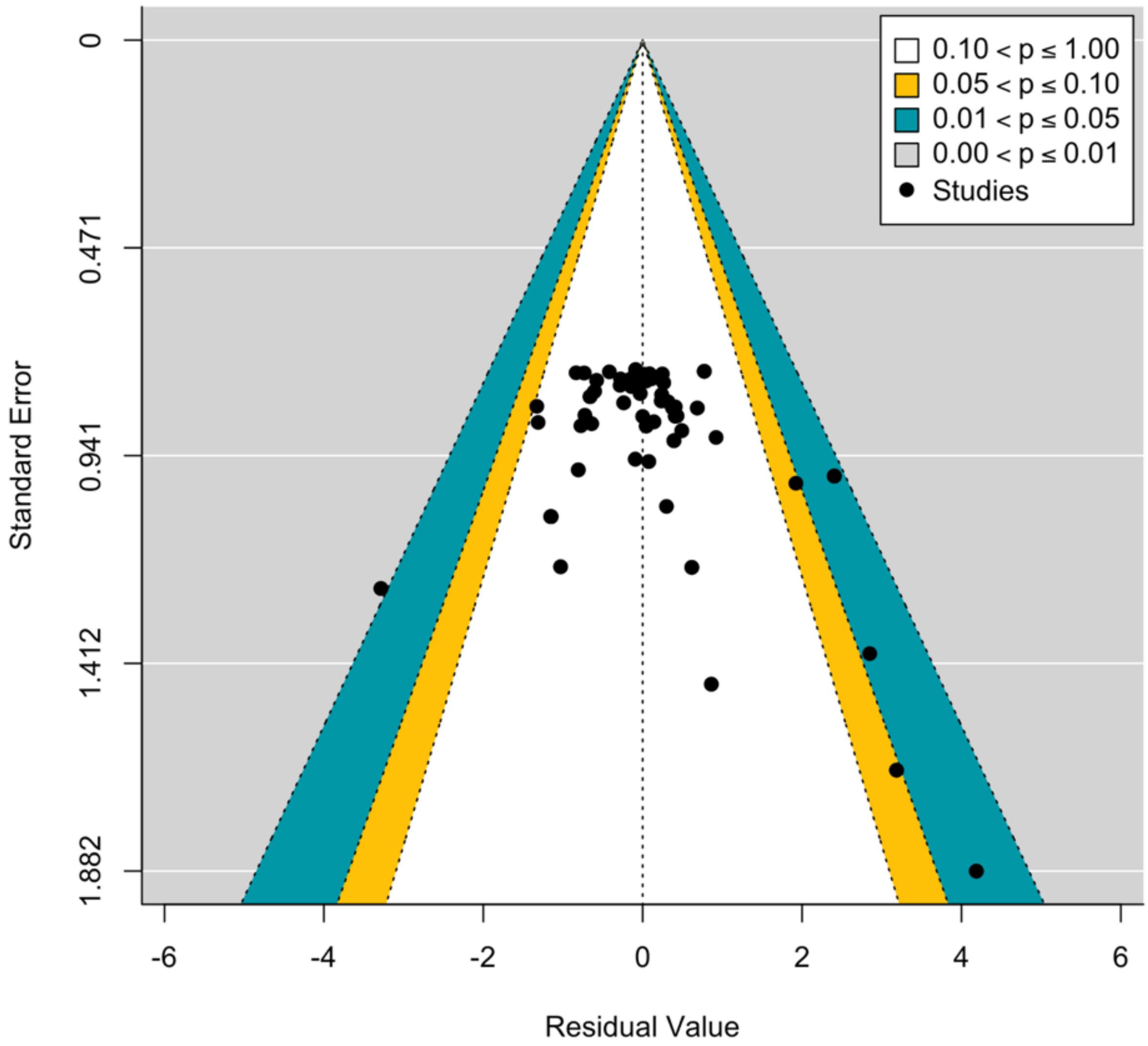

Fig. S4. Funnel plot of glucocorticoid effect size residuals plotted against standard error for intercept-only model. Black points represent individual studies and shaded areas indicate significance of the effect.

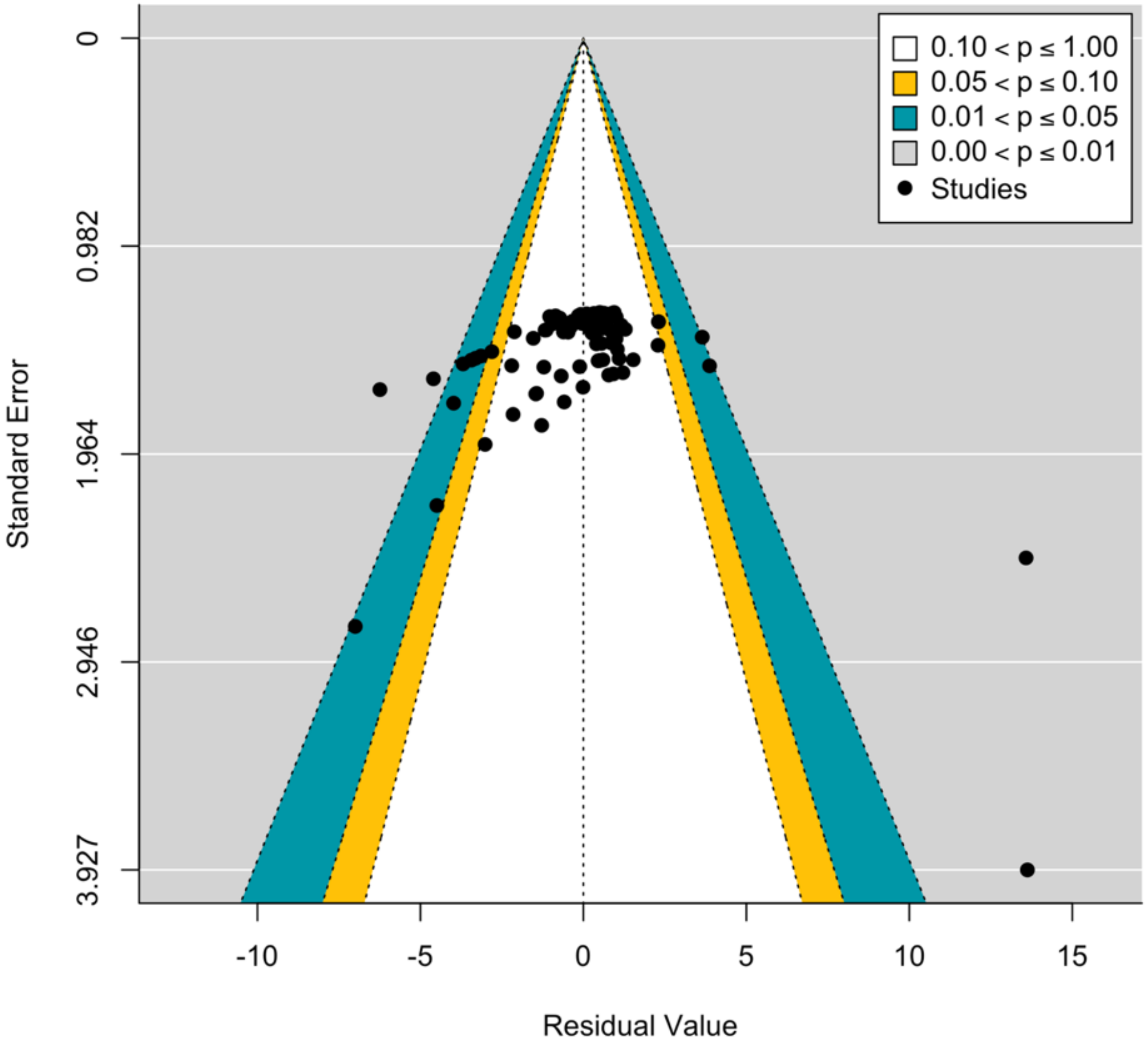

Fig. S5. Funnel plot of fitness effect size residuals plotted against standard error for intercept-only model. Black points represent individual studies and shaded areas indicate significance of the effect.

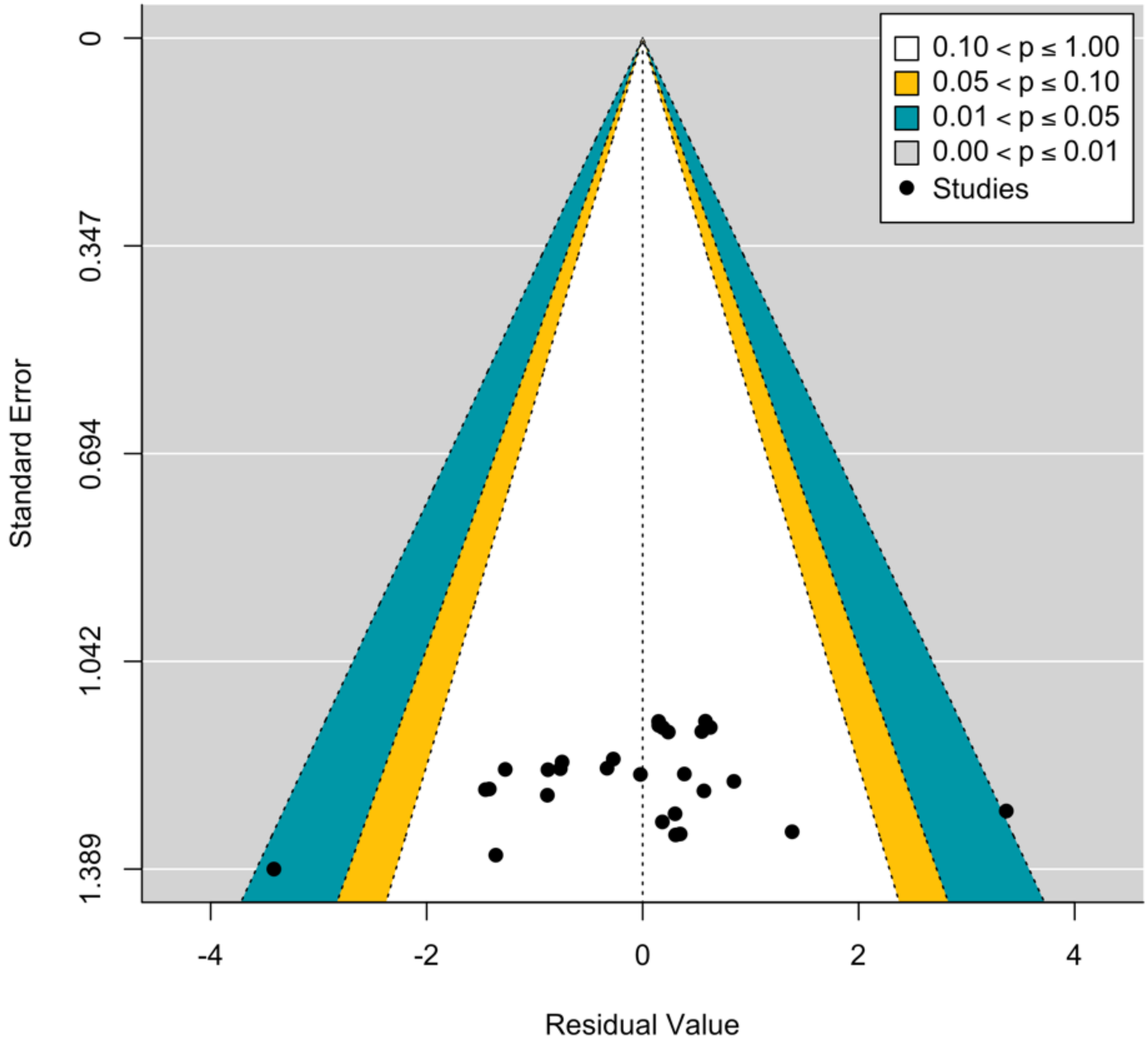

Fig. S6. Funnel plot of fitness effect size residuals plotted against standard error for model with sex as a moderator. Black points represent individual studies and shaded areas indicate significance of the effect.

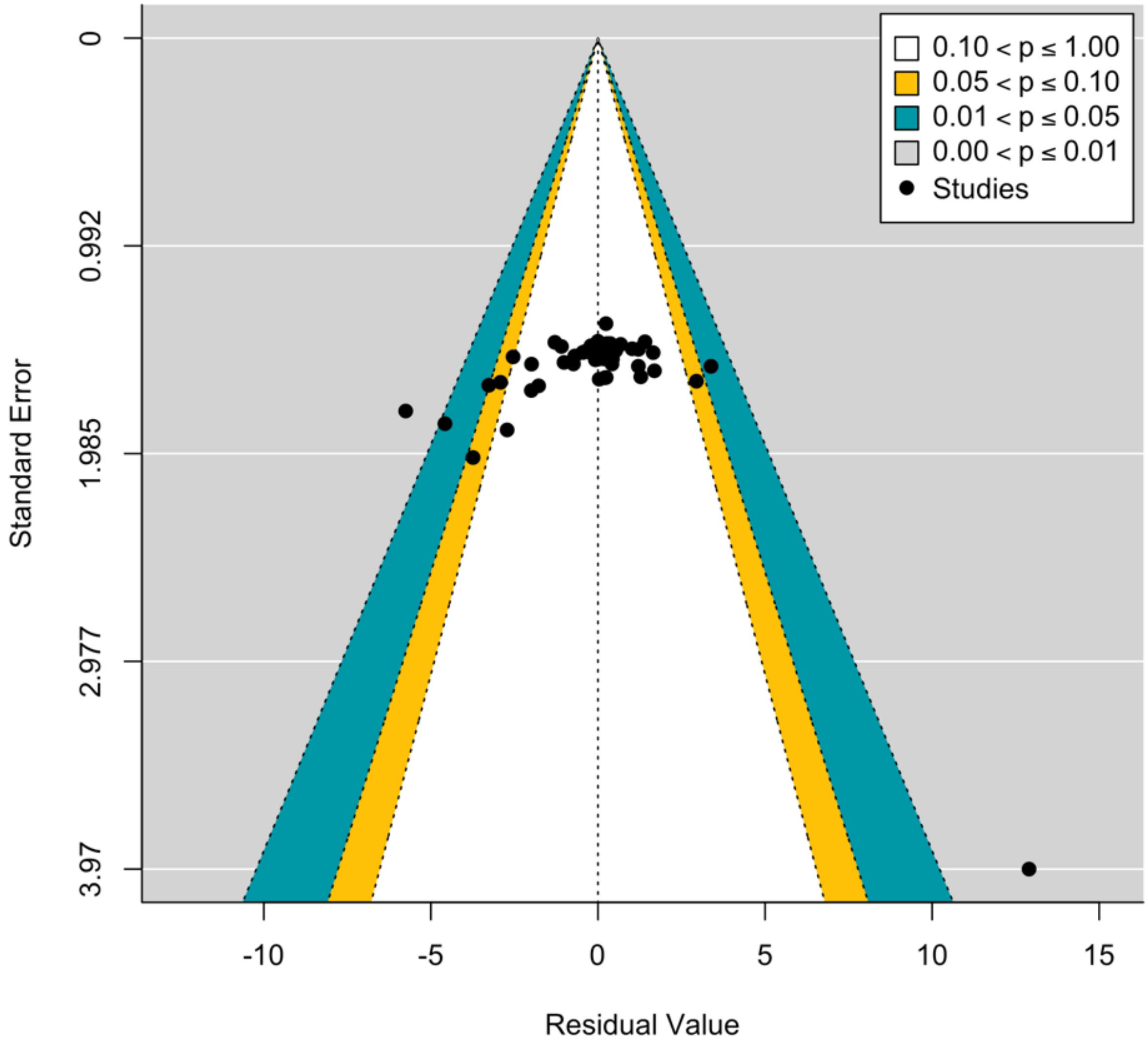

Fig. S7. Funnel plot of fitness effect size residuals plotted against standard error for model with species longevity as a moderator. Black points represent individual studies and shaded areas indicate significance of the effect.

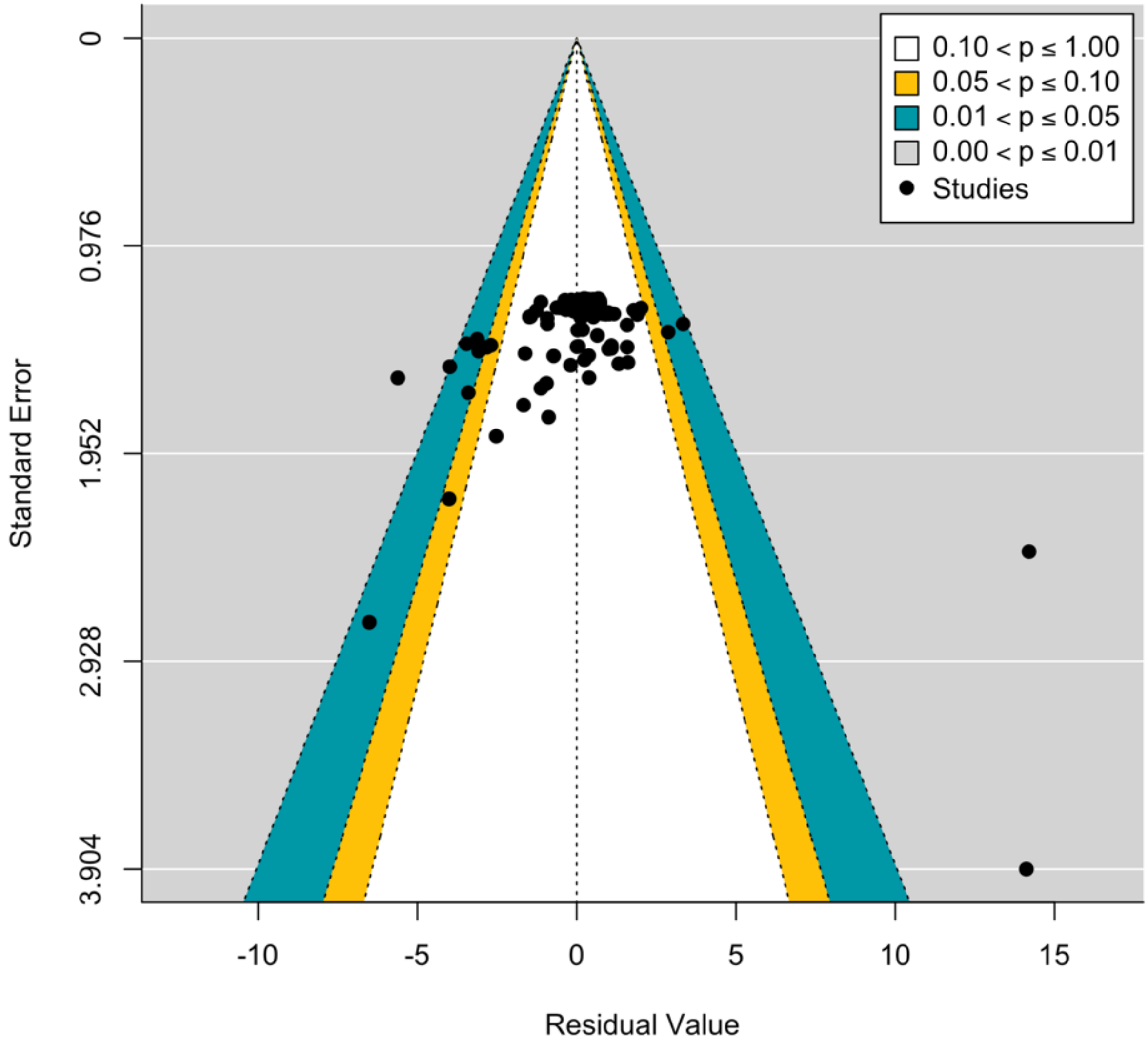

Fig. S8. Funnel plot of fitness effect size residuals plotted against standard error for model with life history stage as a moderator. Black points represent individual studies and shaded areas indicate significance of the effect.

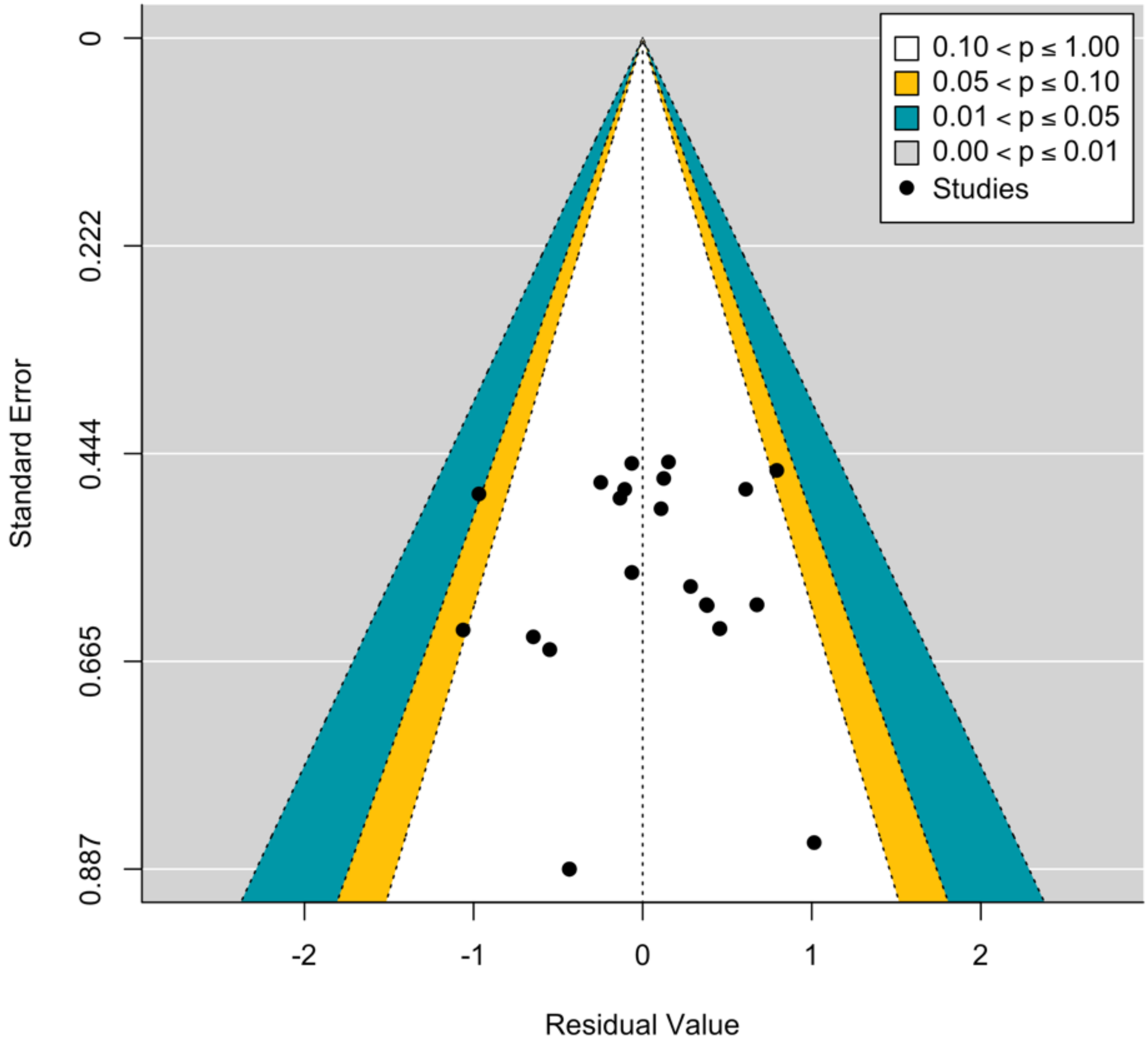

Fig. S9. Funnel plot of glucocorticoid effect size residuals plotted against standard error for model with sex as a moderator. Black points represent individual studies and shaded areas indicate significance of the effect.

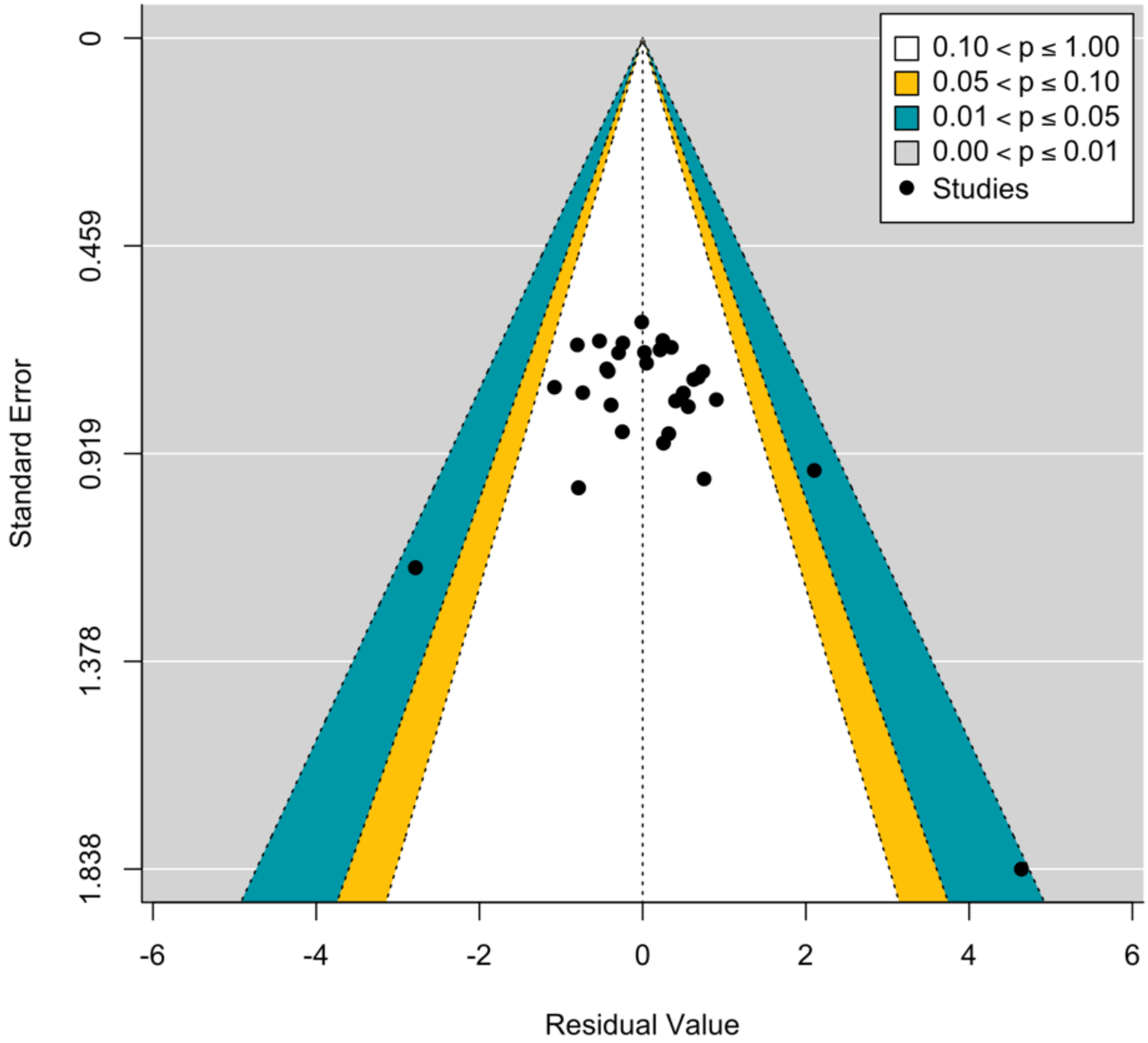

Fig. S10. Funnel plot of glucocorticoid effect size residuals plotted against standard error for model with species longevity as a moderator. Black points represent individual studies and shaded areas indicate significance of the effect.

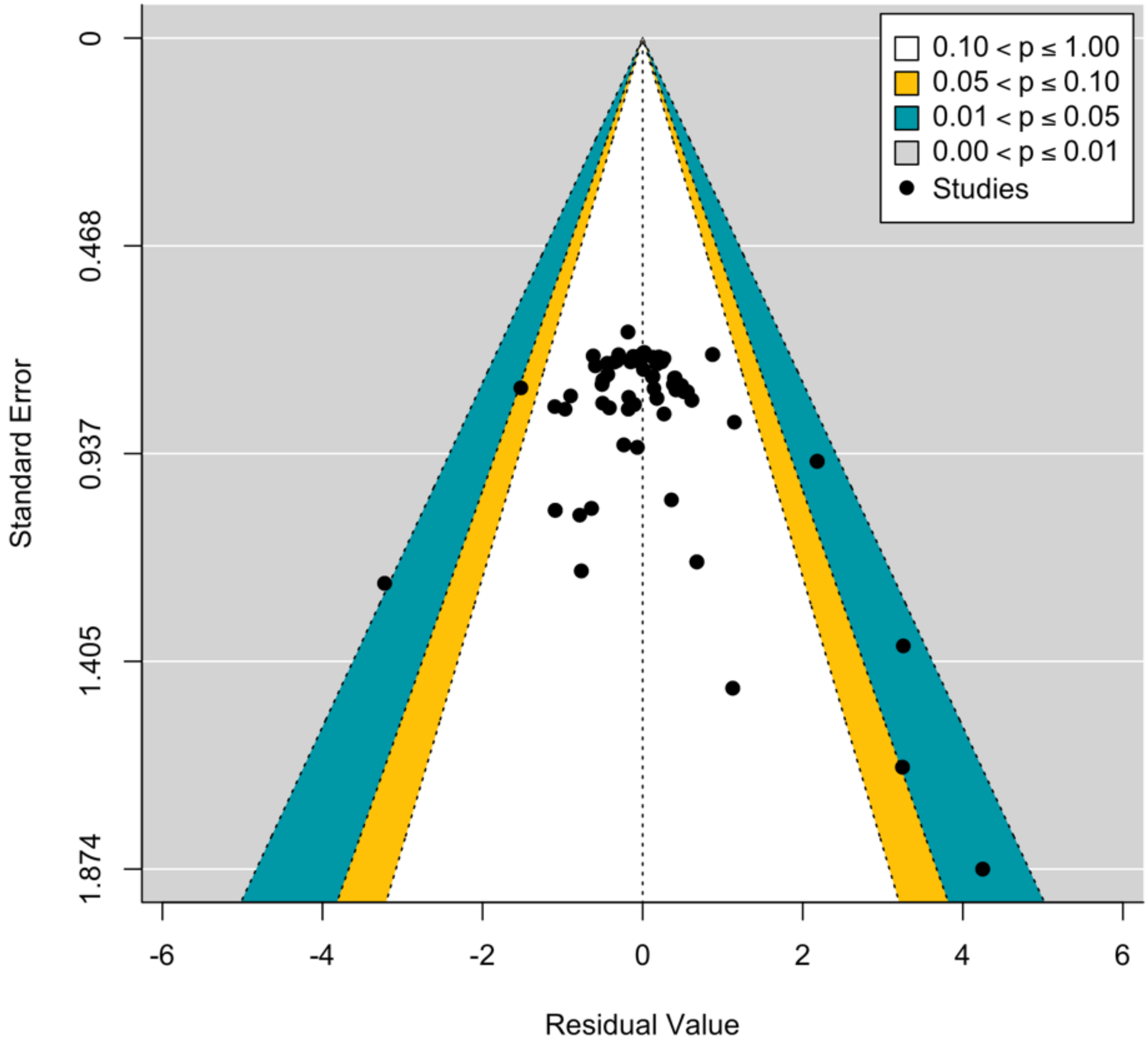

Fig. S11. Funnel plot of glucocorticoid effect size residuals plotted against standard error for model with life history stage as a moderator. Black points represent individual studies and shaded areas indicate significance of the effect.

Supplementary S5. Effects of sample type and sampling invasiveness on heterogeneity in glucocorticoid effect sizes (Table S1).

Table. S1 Pooled mean effect sizes and moderator estimates from meta-regression mixed-effects models testing effects of stressor exposure on glucocorticoid (GC) production with either sample type, sample invasiveness (i.e., whether the sample was collected from the field or directly from the animal), or location of the study (i.e., field or lab) included as a moderator. Both models include random effects for species, stressor type, and individual effect size nested in individual study. Bolded entries indicate significant effects ($p < 0.05$). The references categories for each moderator are blood (sample type model), invasive (invasiveness model), and field (location model).

Moderator	ß (95% CI)	Q-test for heterogeneity (df)	N studies /Effect sizes
Sample type			
FGM	-0.16 (-0.93, 0.60)	**421.82 (104)**	48/109
Water	0.04 (-0.36, 0.44)		
Whole body	-0.55 (-1.56, 0.46)		
Yolk	-0.98 (-2.67, 0.70)		
Invasiveness: non-invasive	-0.13 (-0.80, 0.54)	**432.90 (107)**	48/109
Location: lab	0.38 (-0.10, 0.86)	**435.29 (107)**	48/109
